# The complete mitochondrial genome of the cocoa tussock moth *Orgyia postica* Walker, 1855 (Lepidoptera: Lymantriidae)

**DOI:** 10.1080/23802359.2021.2005474

**Published:** 2021-11-29

**Authors:** Yunbo Duan, Xinghua Hu

**Affiliations:** Guangxi Key Laboratory of Functional Phytochemicals Research and Utilization, Guangxi Institute of Botany, Guangxi Zhuang Autonomous Region and the Chinese Academy of Sciences, Guilin, China

**Keywords:** *Orgyia postica* Walker, mitochondrial genome, phylogenetic analysis

## Abstract

The high-throughput sequencing method was used for the first time to determined complete mitochondrial genome of *Orgyia postica*. The complete mitochondrial genome was a circular molecule with 15,258 bp in full-length, including 13 protein-coding genes (PCGs), 22 transfer RNA genes (tRNAs) and 2 ribosomal RNA genes (rRNAs). The nucleotide composition of *O. postica* mitogenome was A: 37.6%, C: 7.9%, G: 14.8%, T: 39.7%. In addition, the results of the phylogenetic analysis indicate that *O. postica* had the closest relationship with *Laelia suffusa.* Our study will contribute to further research on the evolution of Lymantriidae and the identification of larva species.

## Introduction

*Orgyia postica* Walker, 1855 (Lepidoptera: Lymantriidae) is a crop pest that mainly damages stamens and leaves, It mainly damages more than 70 crops such as tea, cotton, strawberries, loofah, asparagus, radishes, peaches, grapes, pears, citrus, mangoes, causing poor growth, reduced production and even death (Luo, 2016). The sequence of the mitochondrial genome is useful for a deeper understanding of the evolution and identification of insects (Du, 2017; Wang et al., 2019; Chen et al., 2020; Su and Wang, 2020; Dong et al., 2021). Here, we report the complete mitochondrial genome sequence of *O. postica*, in order to better understand the relationship between *O. postica* and related genera, and contribute to understanding the evolution and identification of Lymantriidae.

The sample of *O. postica* was collected at Taxidermy Park in Guangxi University, Nanning, Guangxi Zhuang Autonomous Region, China (22°50′N, 108°17′E). The specimen was deposited at the Guangxi Key Laboratory of Agric-Environment and Agric-Products Safety (Nanning, China) (Voucher specimen number: FDSW202318382-1r) and identified by the head of herbarium Mr Li (Email: lijunlijun1981@163.com). Genomic DNA was extracted using Genomic DNA Kit (TransGen Biotech, Beijing) and constructed the libraries with an average length of 350 bp using the NexteraXT DNA Library Preparation Kit (Illumina, San Diego, CA). Then the libraries were sequenced on Illumina Novaseq 6000 platform, 9.23 Gb clean data was assembled by SPAdes (version 3.11.0) (Bankevich et al., 2012) and annotated by MITOS (http://mitos.bioinf.uni-leipzig.de/index.py). The original sequencing data were uploaded to the NCBI database (https://www.ncbi.nlm.nih.gov/sra/) with the accession number SRR15041082. The complete mitochondrial genome was assigned Genbank accession number MW355619.

The mitochondrial genome of *O. postica* is a closed circular molecule of 15,258 bp, consisting of complex I (NADH dehydrogenase), complex IV (cytochrome C oxidase), ATP synthase, transfer RNAs, ribosomal RNAs and other genes, with the base content of A 37.6%, C 7.9%, G 14.8%, and T 39.7%. The A + T content is 77.3%, showing a strong AT skew. The genome encodes 37 genes, including 13 protein-coding genes (PCGs), 22 transfer RNA genes (tRNAs), and 2 ribosomal RNA genes (rRNAs). With the gene rrnS as a starting point, the gene order of complete mitochondrial is rrnS, trnV-TAC, rrnL, trnL-TAG, ND1, trnS-TGA, CYTB, ND6, trnP-TGG, trnT-TGT, ND4L, ND4, trnH-GTG, ND5, trnF-GAA, trnE-TTC, trnS-GCT, trnN-GTT, trnR-TCG, trnA-TGC, ND3, trnG-TCC, COX3, ATP6, ATP8, trnD-GTC, trnK-CTT, COX2, trnL-TAA, COX1, trnY-GTA, trnC-GCA, trnW-TCA, ND2, trnQ-TTG, trnI-GAT, trnM-CAT. Among these genes, the shortest and longest were 62 (trnR-TCG) and 1692 bp (ND5), and the average gene length was 390.78 bp.

**Figure 1.**
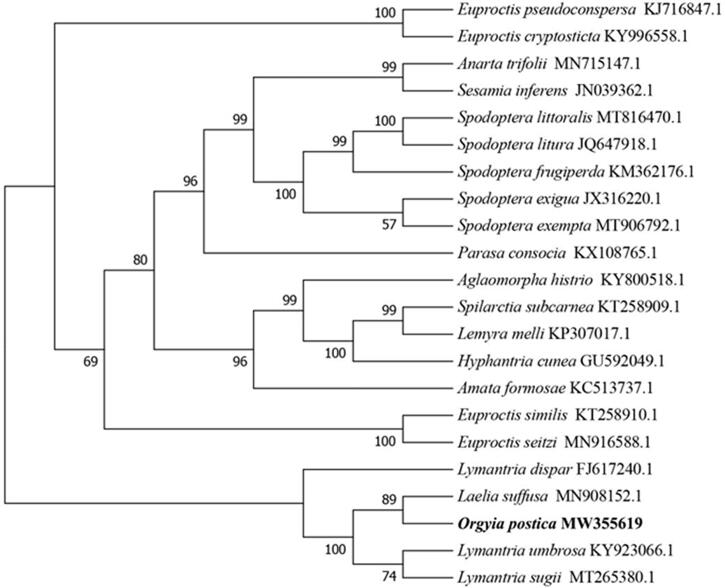
Molecular phylogenetic analysis of *O. postica* and other insects by Maximum Likelihood method. The complete mitochondrial genome was downloaded from NCBI GenBank, with the accession number listed behind the species name. Numbers in each the node indicated the bootstrap support values.

To confirm the phylogenetic position and understand the relationship of *O. postica* within lepidoptera. 21 published complete mitochondrial genome sequences from lepidoptera including *Euproctis pseudoconspersa* (KJ716847.1), *Euproctis cryptosticta* (KY996558.1), *Anarta trifolii* (MN715147.1), *Sesamia inferens* (JN039362.1), *Spodoptera littoralis* (MT816470.1), *Spodoptera litura* (JQ647918.1), *Spodoptera frugiperda* (KM362176.1), *Spodoptera exigua* (JX316220.1), *Spodoptera exempta* (MT906792.1), *Parasa consocia* (KX108765.1), *Aglaomorpha histrio* (KY800518.1), *Spilarctia subcarnea* (KT258909.1), *Lemyra melli* (KP307017.1), *Hyphantria cunea* (GU592049.1), *Amata formosae* (KC513737.1), *Euproctis similis* (KT258910.1), *Euproctis seitzi* (MN916588.1), *Lymantria dispar* (FJ617240.1), *Laelia suffusa* (MN908152.1), *Lymantria umbrosa* (KY923066.1) and *Lymantria sugii* (MT265380.1) species were collected and aligned with *O. postica* by MAFFT7.037 (Katoh and Standley, 2013). The evolutionary history was inferred by using the Maximum Likelihood method with 1000 bootstrap replicates based on the Tamura-Nei model (Tamura and Nei, 1993). The tree with the highest log likelihood (−217,907.0919) is shown. The percentage of trees in which the associated taxa clustered together is shown next to the branches. Initial tree(s) for the heuristic search were obtained automatically by applying Neighbor-Join and BioNJ algorithms to a matrix of pairwise distances estimated using the Maximum Composite Likelihood approach, and then selecting the topology with superior log likelihood value. The tree is drawn to scale, with branch lengths measured in the number of substitutions per site. The analysis involved 22 nucleotide sequences. Codon positions included were 1st + 2nd + 3rd + Noncoding. There was a total of 36,952 positions in the final dataset. Evolutionary analyses were conducted in MEGA7 software version 7.0.18 (Kumar et al., 2016). By using various other families of the order lepidoptera as outgroup we got the final ML tree, then Figure 1 shows that *L. suffusa* has the closest relationship with *O. postica*.

## Data Availability

The genome sequence data that support the findings of this study are openly available in GenBank of NCBI at https://www.ncbi.nlm.nih.gov/ under the accession no. MW355619. The associated BioProject, SRA, and Bio-Sample numbers are PRJNA743722, SRR15041082, and SAMN20058967, respectively.
